# Identification of cuproptosis-related gene clusters and immune cell infiltration in major burns based on machine learning models and experimental validation

**DOI:** 10.3389/fimmu.2024.1335675

**Published:** 2024-02-12

**Authors:** Xin Wang, Zhenfang Xiong, Wangbing Hong, Xincheng Liao, Guangping Yang, Zhengying Jiang, Lanxin Jing, Shengyu Huang, Zhonghua Fu, Feng Zhu

**Affiliations:** ^1^ Medical Center of Burn Plastic and Wound Repair, The First Affiliated Hospital of Nanchang University, Nanchang, Jiangxi, China; ^2^ Department of Critical Care Medicine, Shanghai East Hospital, Tongji University School of Medicine, Shanghai, China; ^3^ Department of Burns, The First Affiliated Hospital, Naval Medical University, Shanghai, China

**Keywords:** cuproptosis, major burns, immune infiltration, molecular clusters, machine learning

## Abstract

**Introduction:**

Burns are a global public health problem. Major burns can stimulate the body to enter a stress state, thereby increasing the risk of infection and adversely affecting the patient’s prognosis. Recently, it has been discovered that cuproptosis, a form of cell death, is associated with various diseases. Our research aims to explore the molecular clusters associated with cuproptosis in major burns and construct predictive models.

**Methods:**

We analyzed the expression and immune infiltration characteristics of cuproptosis-related factors in major burn based on the GSE37069 dataset. Using 553 samples from major burn patients, we explored the molecular clusters based on cuproptosis-related genes and their associated immune cell infiltrates. The WGCNA was utilized to identify cluster-specific genes. Subsequently, the performance of different machine learning models was compared to select the optimal model. The effectiveness of the predictive model was validated using Nomogram, calibration curves, decision curves, and an external dataset. Finally, five core genes related to cuproptosis and major burn have been was validated using RT-qPCR.

**Results:**

In both major burn and normal samples, we determined the cuproptosis-related genes associated with major burns through WGCNA analysis. Through immune infiltrate profiling analysis, we found significant immune differences between different clusters. When K=2, the clustering number is the most stable. GSVA analysis shows that specific genes in cluster 2 are closely associated with various functions. After identifying the cross-core genes, machine learning models indicate that generalized linear models have better accuracy. Ultimately, a generalized linear model for five highly correlated genes was constructed, and validation with an external dataset showed an AUC of 0.982. The accuracy of the model was further verified through calibration curves, decision curves, and modal graphs. Further analysis of clinical relevance revealed that these correlated genes were closely related to time of injury.

**Conclusion:**

This study has revealed the intricate relationship between cuproptosis and major burns. Research has identified 15 cuproptosis-related genes that are associated with major burn. Through a machine learning model, five core genes related to cuproptosis and major burn have been selected and validated.

## 1 Introduction

Burns are one of the most destructive forms of trauma, responsible for more than 265,000 deaths worldwide (1). More than 95% of burns related to fires occur in low-income and middle-income countries, especially in Africa. The high mortality rate associated with major burns is a major cause of loss of disability-adjusted life years (DALYs) in low- and middle-income countries (2) Burns are not just a simple pathophysiological process but rather a destructive injury that leads to structural and functional defects in multiple organ systems. Burns alter metabolic balance, immune responses, and tissue structure, triggering a cascade of physiological responses in patients, including wound infections, respiratory failure, and other illnesses. Physiological and morphological changes vary according to the degree of skin burns, and a high metabolic response can effectively reflect the severity of burns (3). Skin burns can cause physical and mental health problems. Currently, the treatment for major burns mainly focuses on symptomatic supportive care. Studying the pathological mechanisms of burns can help to identify more precise therapeutic targets and help patients recover.

As an essential trace mineral, copper is required for a wide variety of physiological processes in almost all cell types. Cu is an important cofactor in various biological processes, including mitochondrial respiration, biosynthesis, and antioxidant defense (4). It is important to maintain the cellular copper concentration within a relatively low range, because a rapid increase can lead to cellular toxicity. Cuproptosis is a cell death mechanism distinct from classical apoptosis, necroptosis, and ferroptosis (5). Studies have shown that copper-dependent cell death occurs through the direct binding of lipolytic components of the copper-dependent tricarboxylic acid (TCA) cycle (6). Therefore, the intake, distribution, and elimination of Cu are tightly controlled. Furthermore, in humans, the accumulation of Cu and related gene mutations are associated with pathological conditions. Although the concept of cuprotosis was proposed in 2022, related studies have been conducted since several years. Studies have reported that cuprotosis plays a role in signal transduction and regulates the etiology, severity, and progression of cancer. It not only plays a role in chronic diseases but also participates in the occurrence and development of various acute diseases, such as acute myocardial infarction and acute spinal cord injury (7–9). Cuprotosis, an intervention target for copper metabolism disorders, may be a novel approach for treating various diseases. However, the mechanisms underlying the copper surge are not fully understood.

In the present study, we systematically examined the differential expression and immune characteristics of cuproptosis-related genes (CRGs) in healthy individuals and patients with major burns. Based on the expression profiles of the 19 CRGs, we classified 553 patients with major burns into two clusters associated with cuproptosis, and further evaluated the differences in immune cells between the two clusters. Subsequently, using a weighted gene co-expression network analysis (WGCNA) algorithm, we identified cluster-specific coproptosis-related genes and elucidated their diverse biological functions and pathways. By comparing multiple machine-learning algorithms, we established predictive models to determine the risk of different molecular clusters in patients. The performance of the predictive models was validated using a nomogram, calibration curve, decision curve analysis (DCA), and external datasets. Additionally, we investigated the correlation between model-related genes and injury time. Finally, we validated the differential gene expression in the blood of patients with burns using qRT-PCR, providing new insights into predicting the outcome and treatment of major burn clusters and associated risks.

## 2 Materials and methods

### 2.1 Data collection and processing

By accessing the GEO database (10) (https://www.ncbi.nlm.nih.gov/geo/), we obtained the relevant datasets, GSE37069 and GSE19743, and downloaded the series matrix and platform files. The GSE37069 dataset (platform file GPL570) included transcriptome data from 553 blood samples from patients with major burns and 37 samples from healthy participants. The GSE19743 dataset (platform file GPL570) included transcriptome data of 114 blood samples from patients with major burns and 63 from healthy participants. We used the GSE37069 and GSE19743 datasets as the training and validation sets, respectively. By using the “limma” R package for data annotation and normalization, we obtained gene expression files, which provided information related to gene expression.

### 2.2 Differentially expressed genes and CRGs analysis

Based on previous research on cuproptosis, we identified 19 CRGs (11, 12) After obtaining gene expression data, in R software 4.3.0, we used the “limma” package to correct the data. We identified differentially expressed CRGs (DE-CRGs) (P<0.05) between the major burn group and control group. We visualized the significant DE-CRGs using the “Heatmap” package in the form of a heatmap, and generated heatmap and box plots.

### 2.3 Analysis of immune cell infiltration

After obtaining gene expression data, the CIBERSORT algorithm was applied to perform immune cell infiltration analysis (13). Analysis included 22 types of immune cells. Through Monte Carlo sampling, the infiltrating expression levels of immune cells were obtained, and samples with a p-value <0.05 are considered as accurate immune cell expression levels. The obtained gene expression levels were relative expression levels, and the sum of the expression levels of all genes equals 1.

### 2.4 Immune cell differential analysis and its correlation with CRGs

After completing the immune cell infiltration analysis, differential analysis of immune cell infiltration was performed based on the files obtained (P<0.05). To understand the relationship between CRGs and immune cells in major burns, Spearman correlation coefficient was used for correlation analysis, considering *P*<0.05 as significant. The visualization of the results was accomplished using the “ggplot2” package and producing heat maps.

### 2.5 Unsupervised clustering analysis of major burn samples

By running the “ConsensusClusterPlus” package and using the K-means algorithm, we performed unsupervised clustering analysis of 533 blood samples from patients with major burns. We selected the maximum number of subtypes K (K=9) and obtained consistent clustering scores (14). Based on the cumulative distribution function (CDF) curve and clustering consistency score, the optimal number of clusters was selected for further analysis.

### 2.6 Gene set variation analysis and weighted gene co-expression network analysis

In order to understand the expression differences of CRGs in different clustering gene sets and pathways, GSVA enrichment analysis was performed on different genes using the “GSVA” software package (15). The “limma” R software package was used to score the GSVA results, and the scores were adjusted. After performing pathway differential analysis, pathways with a p-value <0.05 were retained. Finally, the top ten pathways with the most prominent upregulation and downregulation were selected. Disease-related modules were identified using WGCNA, and the genes within these modules were identified as key genes for the disease (16–18). By using the ‘WGCNA’ package in R, we selected the top 25% of genes with the highest variability for WGCNA. We checked for missing values, performed sample clustering, and obtained a cluster heatmap. We then obtained a scatter plot of power values and a scatter plot of the fit index with power values, as well as the average connectivity with power values. Gene clustering was conducted using the TOM (Topological Overlap Measure) matrix with the module gene number set to 100, allowing for dynamic module identification. We generated a heatmap of the module genes and obtained a heatmap of the correlations between the gene modules and traits. Subsequent analyses were performed on the gene modules with high correlation coefficients and low *p* - values.

### 2.7 Obtaining hub genes

Through previous WGCNAs, we obtained hub genes for major burns and cluster analysis. The “Venn Diagram” R software package was used to perform clustering analysis on the intersection of hub genes from the disease to obtain the hub genes within the intersection. A Venn diagram was used to visualize the hub genes, and a hub gene list was created for further analysis.

### 2.8 Establishment of machine learning models

We built multiple machine-learning models, including Random Forest (RF), Support Vector Machine (SVM), Generalized Linear Model (GLM), and Extreme Gradient Boosting (XGBoost)(19). RF is an ensemble learning algorithm that consists of multiple decision trees. It can manage a large number of features and samples, and is robust against missing values and outliers (20). SVM is a common supervised learning algorithm used for classification and regression problems (21, 22). Generalized Linear Model establishes a linear function between the response variable and a set of explanatory variables using a nonlinear link function to capture the nonlinear relationship of the response variable. It can handle different types of response variables, and is suitable for diverse data analysis problems. XGBoost is a machine-learning algorithm that combines multiple weak learners (usually decision trees) to create a powerful learning model. Each weak learner gradually fits the negative gradient of the error using a gradient descent (23, 24). XGBoost incorporates techniques such as cross-validation, regularization, and pruning to improve model generalization and control overfitting (25, 26). For the aforementioned analysis, the CRGs of the two clusters were obtained. The “caret” R package was used for analysis. The data were divided into training (70%) and testing (30%) sets. The machine learning model was built using the training set data, the prediction function was defined, and visualization was performed, including residual cumulative distribution plots and boxplots for the four models. Next, the “pROC” R package was used to plot ROC curves for the four methods. Residual box plots and ROC curves were used to determine the best model. Gene importance scores were calculated using the four methods, and the top five genes were selected as hub genes for major burns in the best model.

### 2.9 Column line chart and model validation

Using the “rms” R package, analyze gene expression files to obtain a gene list, and subsequently visualize the results by creating a column line chart. The column-line chart scores the expression of each gene and generates the total score. The probability of disease occurrence was predicted based on the total score. Decision curves and decision curve analyses (DCA) were used to evaluate the accuracy of the model.

### 2.10 Independent validation analysis

Similarly, the validation dataset, GSE19743 (GPL570), was divided into groups. The training group comprised 70% of the data while the test group comprised the remaining 30%. The appropriate model selected from earlier is used, and the “pROC” R package is employed to visualize the results and evaluate the accuracy of the model on the validation dataset. In addition, we analyzed the relationship between disease-related gene features and injury time.

### 2.11 Real-time quantitative polymerase chain reaction

Blood samples were collected from patients with major burns and healthy controls. The RNA was extracted using a blood leukocyte protein extraction kit (Bestbia, Shanghai, China). The RNA was then reverse-transcribed into cDNA using the SweScript RT 1 First Strand cDNA Synthesis Kit (with gDNA Remover) (Servicebio, Wuhan, China) at a concentration of 3000 ng. For qRT-PCR analysis, 2 × Universal Blue SYBR Green qPCR Master MIX (Servicebio, Wuhan, China) and the StepOnePlus Real-Time System (Applied Biosystems, Marsiling, Singapore) were used. The expression levels of target genes were normalized to β-actin. We determined the relative gene expression levels through the 2-ΔΔCt method. The primer sequences are as follows: Forward 5′- TCTCCCAAGTCCACACAGG-3′ and reverse 5′- GGCACGAAGGCTCATCA -3′ for human β-actin, forward 5′- CACAGGAGCAAAAGTCGGGACA-3′ and reverse 5′- GTGTCTTCACTCTGCTTTTCTCG -3′ for human LUC7L3, forward 5′- TCCGATAGCGAGGTGGTGCGG -3′ and reverse 5′- TGGAGTGACCTGGCATGTGCAT -3′ for human MBLAC2, forward 5′-GTAACAAGTGCCACCAGTCTGC-3′ and reverse 5′- TGTCCAGAGACTGCATCGGCTT -3′ for human LRRC47, forward 5′- GAACTACGGAAAGCCGAAAAGGC -3′ and reverse 5′- CCTTCAGCTGTGCAGAATGCTC -3′ for human OFD1, forward 5′- AATGTCATCCCTGAGTGGCACC-3′ and reverse 5′- GCAAGTCATTCTGTGGTAAGCCT -3′ for human USPL1.

### 2.12 Ethics statement

The study design was approved by the Committee of Clinical Ethics of Nanchang First Hospital [ethics number: (2023) CDYFYYLK (08-029)]. All clinical experimental procedures were conducted in accordance with Ethics Committee regulations.

### 2.13 Statistical analysis

GraphPad Prism 8.0 software (San Diego, CA, USA) was used for graphing, calculations, and statistical analysis of the qRT-PCR results. Student’s t-test was used to compare the mean values of different groups. Statistical significance was set at p < 0.05.

## 3 Results

### 3.1 DE-CRGs expression analysis in major burn

**Figure 1:**
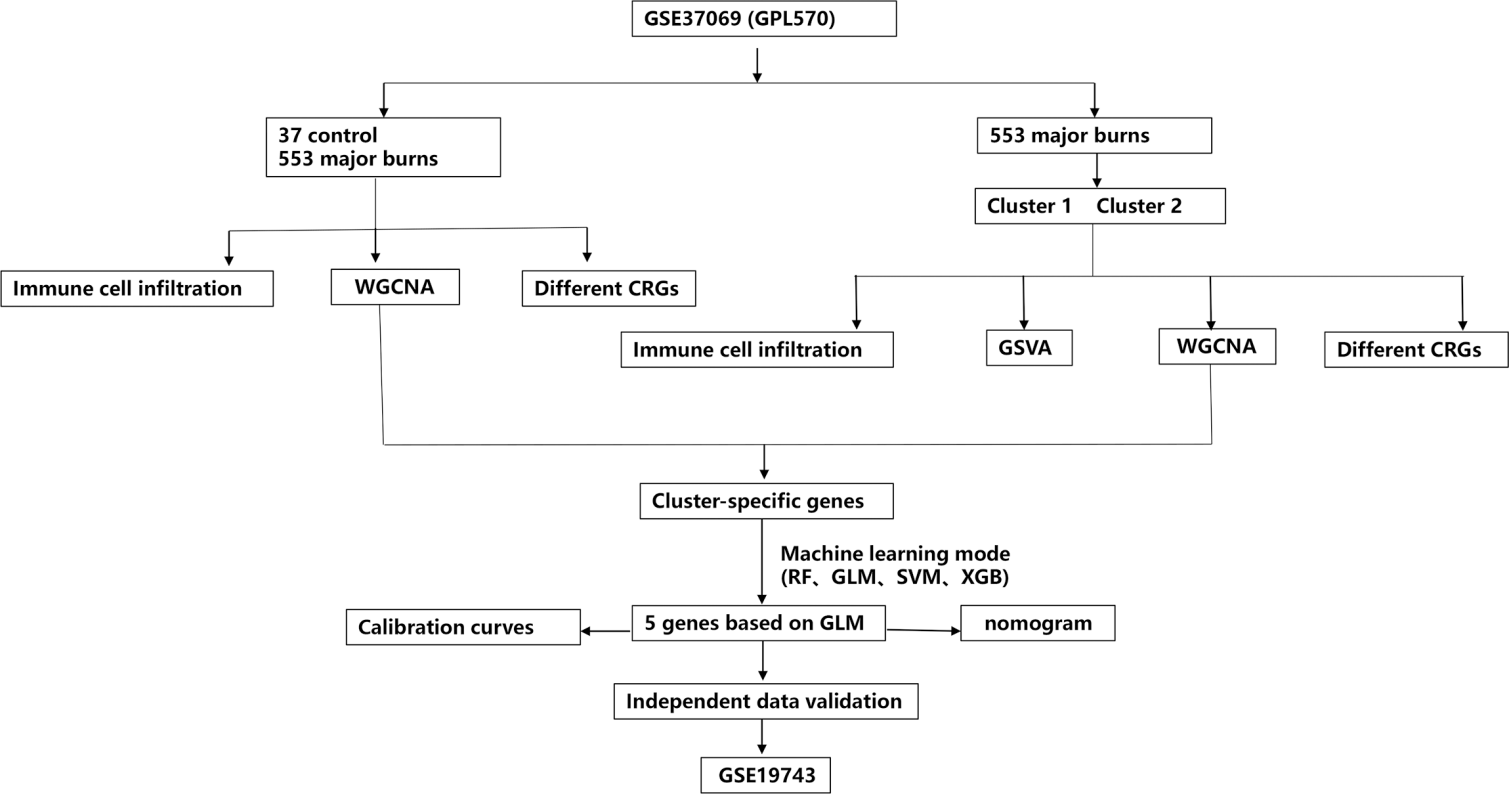
The study flow chart.

**Figure 2:**
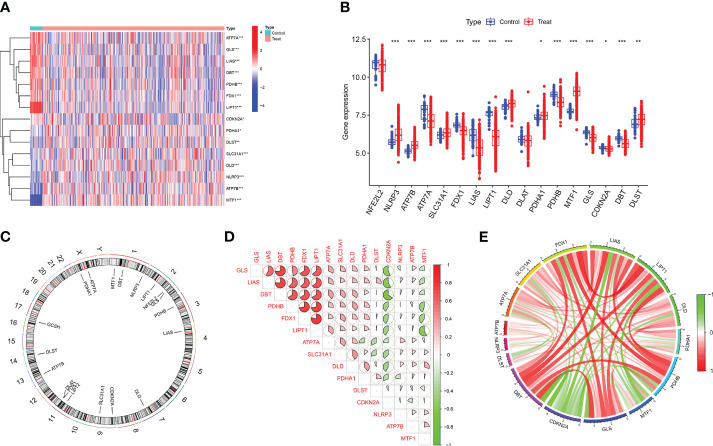
Identification of differentially expressed CRGs in patients with major burn. **(A)** The expression patterns of 15 DE-CRGs were presented in the heatmap. **(B)** Boxplots showed the expression of 17 major burn-related CRGs between the control group and the major burn group. **(C)** The location of 19 CRGs on chromosomes. **(D)** Correlation analysis of 15 differentially expressed CRGs. Red and green colors respectively represent positive and negative correlations. The correlation coefficients were marked with the area of the pie chart. **(E)** Gene relationship network diagram of 15 differentially expressed CRGs. (*p<0.05, **p<0.01, ***p<0.001).

In this study, the “Limma” R package was used to identify differentially expressed CRGs in the dataset GSE37069. A detailed flowchart of the research procedure is shown in **Figure 1**. The selection standard was set at |log2 FC| > 1 and *p*-value < 0.05. Box plots and heat maps (**Figures 2A–C**) were generated to visualize differential gene expression. Among the 19 CRGs, 17 CRGs were associated with major burns and 15 CRGs showed significant differential expression, with seven genes downregulated (ATP7A, GLS, LIAS, DBT, PDHB, FDX1, and LIPT1) and eight upregulated genes (CDKN2A, PDHA1, DLST, SLC31A1, DLD, NLRP3, ATP7B, and MTF1). Subsequently, correlation analysis was performed on these differentially expressed CRGs to explore the interactions between the cuproptosis regulatory factors. Surprisingly, some cuproptosis-related regulatory factors, such as LIPT1, FDX1, PDHB, DBT, LIAS, and GLS, exhibited strong synergistic effects. However, CDKN2A exhibited strong antagonistic effects against DBT, LIAS, and GLS. The gene network is depicted in the figure (**Figures 2D, E**).

### 3.2 Analysis of immune cell infiltration in patients with major burns

**Figure 3:**
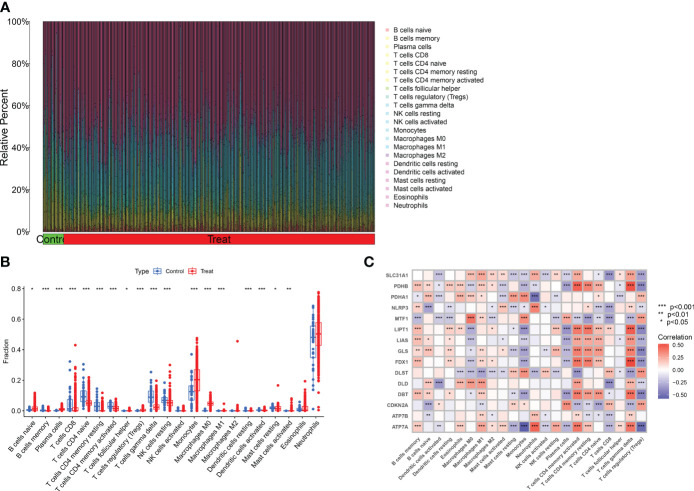
Analysis of immune cell infiltration in patients with major burn. **(A)** CIBERSORT analysis revealed differences in the abundance of 22 infiltrating immune cell types between the major burn and control groups. **(B)** Boxplots showed the differences in immune infiltrating between major burn and control groups. **(C)** correlation analysis between 15 DE- CRGs and infiltrated immune cells. *p<0.05, **p<0.01, ***p<0.001.

To analyze the changes in immune cells in patients with major burns, we performed a differential analysis of immune cell infiltration. The results showed elevated expression levels of naïve B cells, plasma cells, regulatory T cells (Tregs), monocytes, macrophages M0, eosinophils, and neutrophils among the 22 immune cell types (**Figures 3A, B**) in major burns. This suggests that immune cells play a role in disease progression after major burns. Furthermore, we performed correlation analysis and found that several CRGs were closely correlated with immune cells (**Figure 3C**). For example, ATP7A was significantly associated with memory B cells, eosinophils, macrophages M1, activated mast cells, resting mast cells, monocytes, neutrophils, activated NK cells, resting NK cells, plasma cells, CD4 memory activated T cells CD4 memory resting, T cells CD8, T cells, gamma delta T cells, and regulatory T cells (Tregs) (P<0.0001).

### 3.3 Identification of cuproptosis clusters in major burns

**Figure 4:**
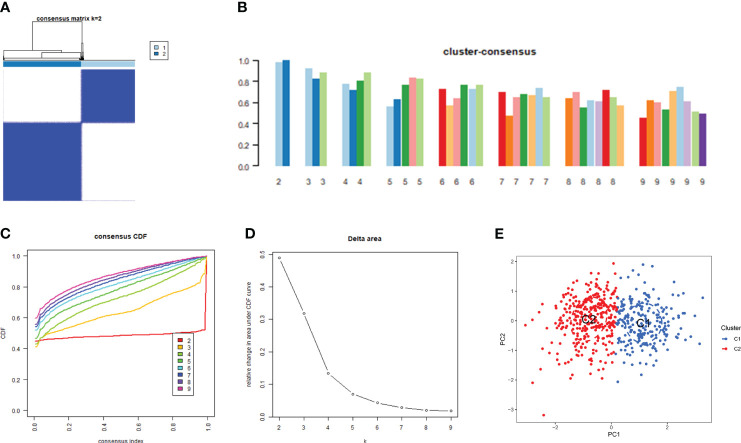
Identification of cuproptosis-related molecular clusters in major burns. **(A)** Consensus clustering matrix when k = 2. **(B–D)** the score of consensus clustering **(B)** Representative cumulative distribution function (CDF) curves **(C)**, CDF delta area curves **(D)**. **(E)** PCA visualizes the distribution of two subtypes.

Consensus clustering is a component of precision medicine and systems biology used to define patient populations or biological molecules. Consensus clustering is a widely used integrated approach that combines outputs from multiple runs of nondeterministic clustering algorithms (27, 28). We applied CRGs to group samples of patients with major burns and found that when the value of k was 2 (k = 2), clustering was the most stable (**Figure 4A**). The CDF curve showed minimal fluctuations within the range of consistency index from 0.2 to 0.9 (**Figures 4C, D**). Furthermore, when k = 2, the consistency scores for each subtype reached their highest values (**Figure 4B**). PCA results indicated significant differences between these two clusters (**Figure 4E**).

### 3.4 Differentiation of cuproptosis regulators and immune infiltration between the cuproptosis clusters

**Figure 5:**
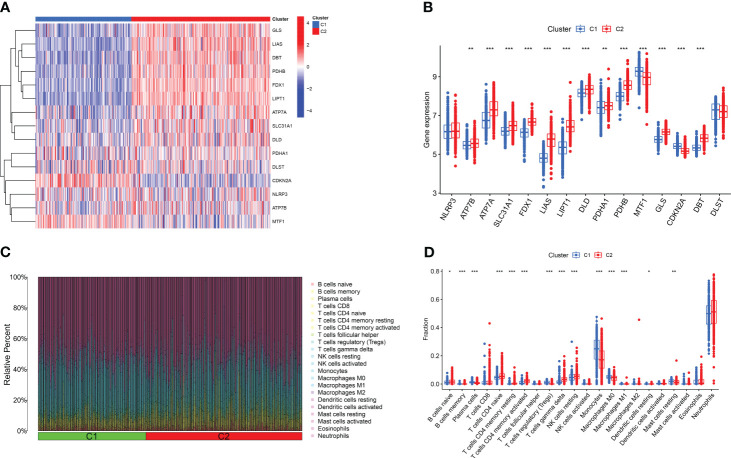
Comparison of CRGs expression and immune cell infiltration between molecular subtypes of major burns. **(A)** Distinct CRGs expression profiles were observed between Cluster 1 and Cluster 2. **(B)** Boxplots showed the expression of 15 CRGs between two cuproptosis clusters. **(C)** The difference in the abundance of 22 infiltrating immune cell types between the two clusters. **(D)** Boxplots showed the differences in immune infiltrating between two cuproptosis clusters. *p<0.05, **p<0.01, ***p<0.001.

Based on the 15 DE-CRGs, we conducted a differential analysis of clusters 1 and 2 to study the expression levels of CRGs and immune cell infiltration in both clusters. The results showed that MTF1, and CDKN2A were highly expressed in cluster 1, whereas ATP7B, ATP7A, SLC31A1, FDX1, LIAS, LIPT1, DLD, PDHA1, PDHB, GLS, and DBT were highly expressed in cluster 2 (**Figures 5A, B**). In terms of immune cell infiltration analysis, there were also significant differences in the levels of immune cell infiltration between Clusters 1 and 2. Cluster 1 had relatively higher expression levels in plasma cells, T cells regulatory (Tregs), monocytes, macrophages M0 and resting mast cells resting, whereas cluster 2 had relatively higher expression levels in B cells naïve, T cells CD4 naïve, T cell CD4 memory resting, T cells CD4 memory activated, T cells gamma delta, resting NK cells resting, and Neutrophils (**Figures 5C, D**).

### 3.5 Biological characteristics between two cuproptosis clusters

**Figure 6:**
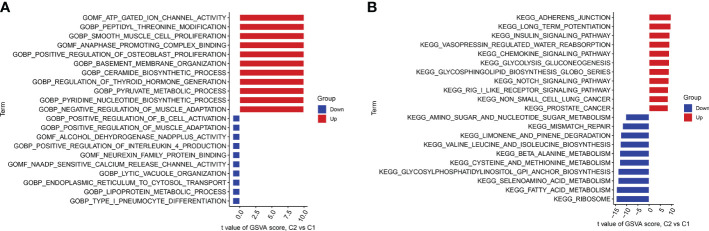
GSVA t-value ranking differences in biological characteristics between two cuproptosis clusters. **(A)** Differences in biological functions between Cluster1 and Cluster2 samples ranked by t-value of GSVA method. **(B)** Differences in hallmark pathway activities between Cluster1 and Cluster2 samples ranked by t-value of GSVA method.

We performed GSVA enrichment analysis on different CRGs using the “GSVA” software package. We used the “limma” R package to score the GSVA results and adjusted the scores accordingly. After conducting pathway differential analysis, pathways with a p-value < 0.05 were retained. Finally, we selected the top 10 pathways with the most significant upregulation and downregulation. Functional enrichment results revealed that ATP ion channel activity and smooth muscle cell proliferation were significantly upregulated in cluster 2, whereas the lipid metabolism process and B cell positive regulation were significantly upregulated in cluster 1 (**Figure 6A**). Kyoto Encyclopedia of Genes and Genomes (KEGG) enrichment analysis revealed that the ribosome pathway showed the most significant upregulation in cluster 1, whereas the adherens junction pathway showed the most significant upregulation in cluster 2 (**Figure 6B**).

### 3.6 Gene modules screening and co-expression network construction

**Figure 7:**
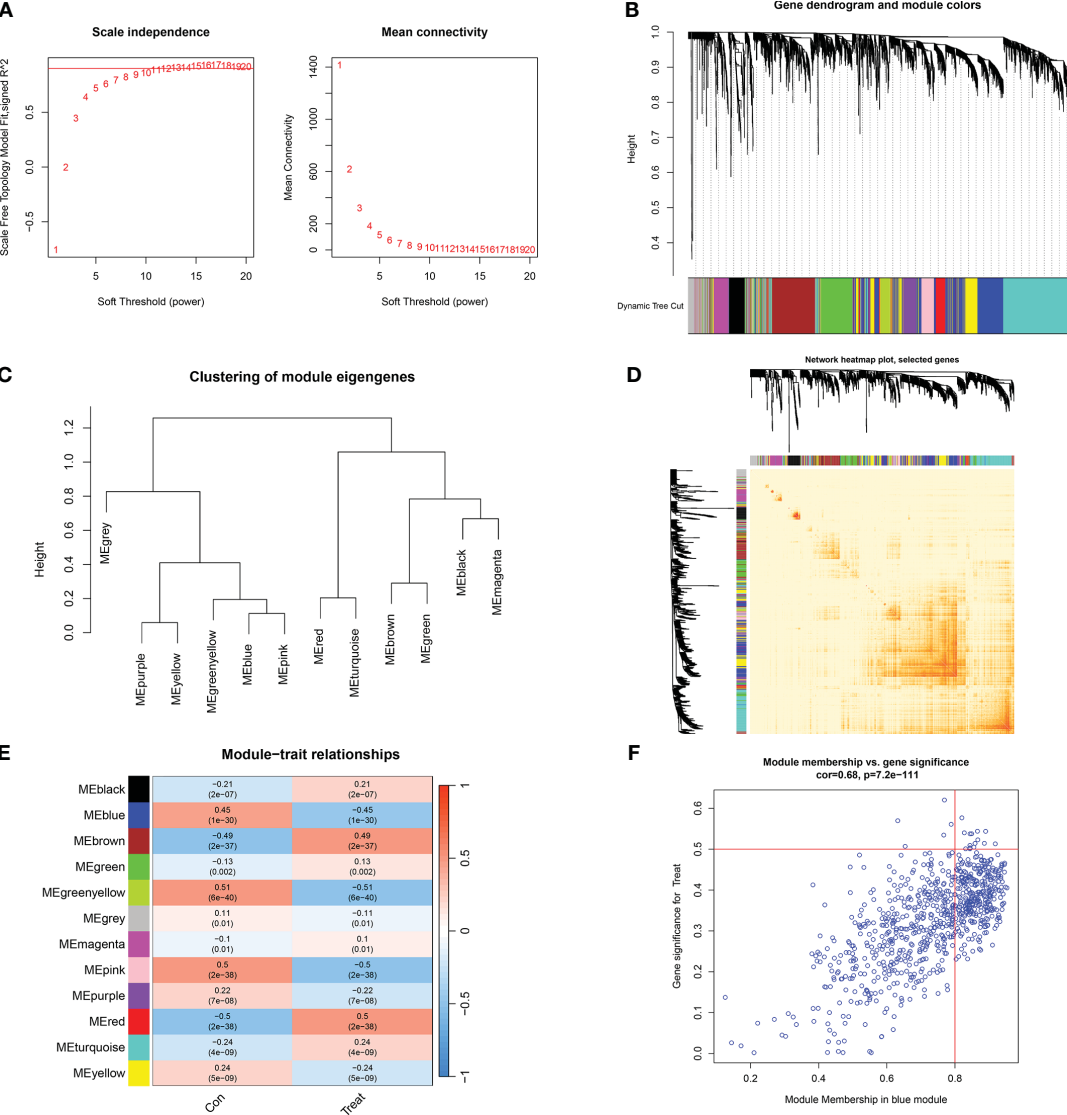
Co-expression network of differentially expressed genes in major burns. **(A)** Selection of soft threshold power. **(B)** Dendrogram of co-expression module clustering. **(C)** Representative clustering of module characteristic genes. **(D)** Representative heat map of correlations between 12 modules. **(E)** Correlation analysis between module characteristic genes and clinical states. Each row represents a module, and each column represents a clinical state. **(F)** Scatter plot of module membership in the blue module and significance of genes in major burns.

We established co-expression networks and modules using the WGCNA algorithm in normal individuals and patients with major burns to identify key gene modules related to major burns. The variance in gene expression in the GSE37069 dataset was evaluated, and the top 25% of genes with the highest variance were selected for further analysis. Co-expression gene modules were identified when the soft threshold was set to 10 and the scale-free topology fit index (R^2^) was 0.9, co-expression gene modules were identified (**Figure 7A**). Twelve different color-coded co-expression modules were dynamically obtained, and a heatmap of the topological overlap matrix (TOM) was generated (**Figures 7B–D**). The blue module comprised 809 genes closely associated with major burns, including 15 core genes (**Figure 7E**). In the gene module, there was a strong positive correlation between the blue module and genes related to the module (**Figure 7F**).

**Figure 8:**
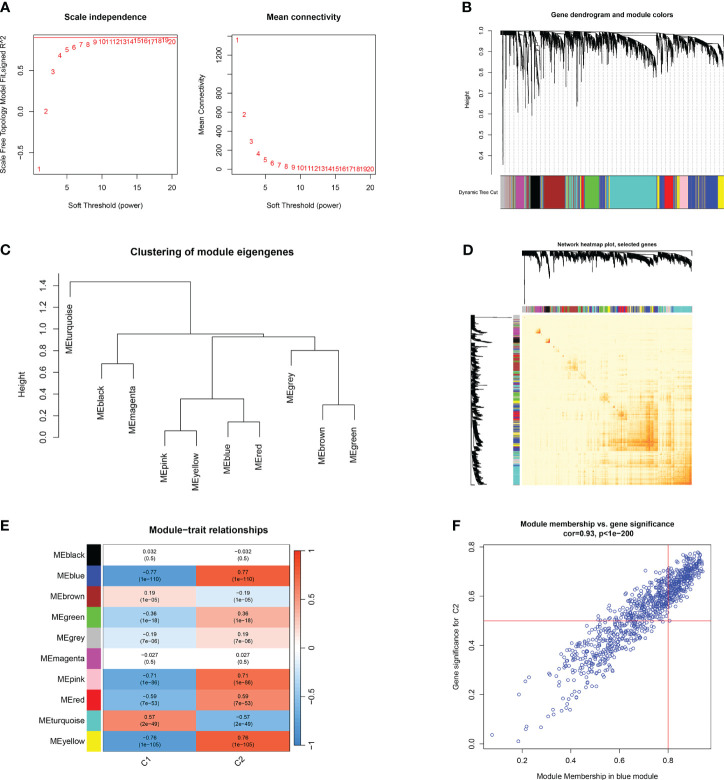
Co-expression network of differentially expressed genes between two clusters of CRGs. **(A)** Selection of soft threshold power. **(B)** Dendrogram of co-expression module clustering. **(C)** Representative clustering of module characteristic genes. **(D)** Representative heat map of correlations between 10 modules. **(E)** Correlation analysis between module characteristic genes and clinical states. Each row represents a module, and each column represents a clinical state. **(F)** Scatter plot of module membership in the blue module and significance of genes in Cluster1.

**Figure 9:**
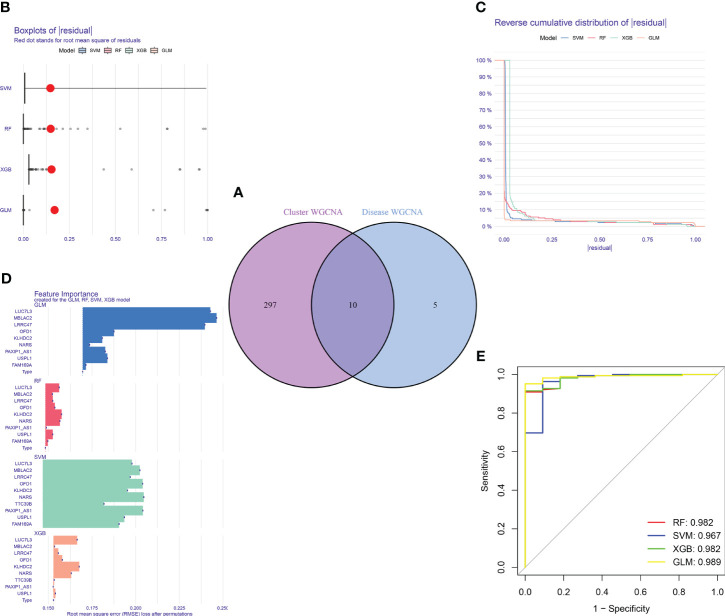
Construction and evaluation of RF, SVM, GLM, and XGB machine models. **(A)** Identification of the intersected genes of disease WGCNA and cluster-WGCNA. The intersection of hub genes in the two modules yielded 10 genes. **(B)** Cumulative residual distribution of each machine learning model. **(C)** Boxplots showed the residuals of each machine learning model. Red dot represented the root mean square of residuals (RMSE). **(D)** The important features in RF, SVM, GLM, and XGB machine models. **(E)** ROC analysis of four machine learning models based on 5-fold cross-validation in the testing cohort.

Furthermore, the WGCNA algorithm was used to analyze the key gene modules closely related to CRGs clustering. We selected β = 10 and R^2^ = 0.9 as the most suitable soft-thresholding parameters to construct the scale-free network (**Figure 8A**). Specifically, 10 modules were identified as significant, and the heat map illustrates the TOM of all module-related genes (**Figures 8B–E**). Analysis of module-clinical features (Clusters 1 and 2) revealed a high correlation between the blue module (926 genes) and major burn clusters, including 307 core genes (**Figure 8F**). Finally, the core genes intersecting between the two modules were obtained using the “Venn” package in R, resulting in 10 genes (**Figure 9A**).

### 3.7 Construction of machine learning models

For the purpose of further identifying subtype-specific genes with high diagnostic value, we generated expression profiles of ten cluster-specific DEGs from the major burn training cohort and established four validated machine learning models (RF, SVM, GLM, XGBoost). The “DALEX” package was used to interpret the four models and plot the residual distribution of each model on the test set. The residual values of the four machine-learning models were relatively low (**Figures 9B, C**). Subsequently, the top ten significant feature variables for each model were ranked based on the root mean square error (RMSE) (**Figure 9D**). The discriminative performance of the four machine learning algorithms was assessed by performing 5-fold cross-validation on the training set (GSE37069 dataset) and calculating the receiver operating characteristic (ROC) curves (**Figure 9E**). The areas under the ROC curve (AUC) for the four models were as follows: RF (AUC = 0.982), SVM (AUC = 0.967), XGB (AUC = 0.982), and GLM (AUC = 0.989). Based on the residual and AUC values, the GLM machine learning model exhibited the best performance in distinguishing between different clusters of patients with major burns. The top five genes (LUC7L3, MBLAC2, LRRC47, OFD1, and USPL1) ranked in the GLM were selected as predictive genes for further analyses.

### 3.8 Assessment of machine learning models

**Figure 10:**
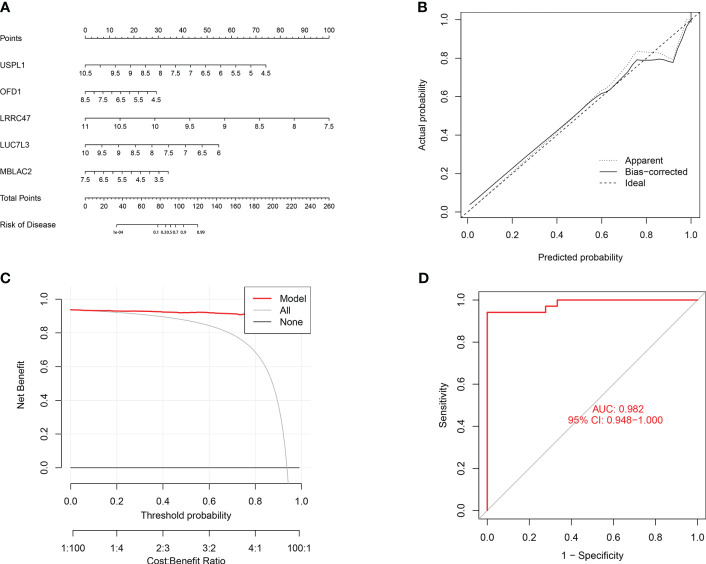
Validation of the 5-gene-based GLM model. **(A)** Construction of a nomogram for predicting the risk of major burn clusters based on the 5-gene-based GLM Model. **(B, C)** Construction of calibration curve **(B)** and DCA **(C)** for assessing the predictive efficiency of the nomogram model. **(D)** the ROC curve of the five genes of the GLM model. the ROC curve of the five genes of the GLM model exhibited good performance (AUC= 0.982).

**Figure 11:**
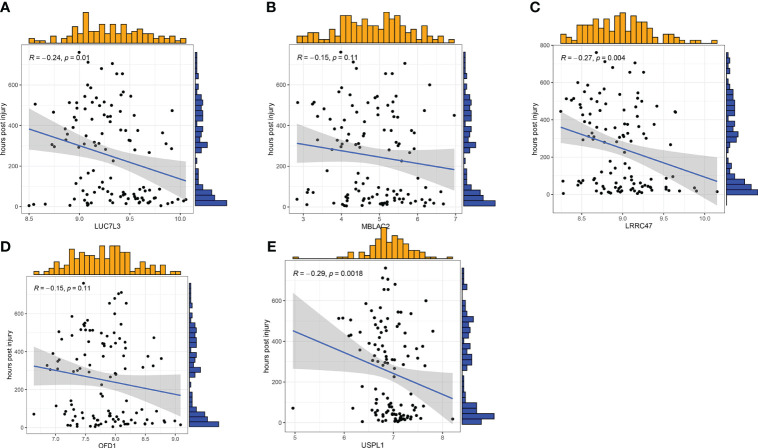
Correlation analysis between gene expression and disease status in an independent dataset of patients with major burn. **(A–E)** Correlation between the 5 genes and active/latent major burn. LUC7L3, LRRC47 and USPL1 were negatively correlated with major burn.

To further assess the predictive efficiency of the GLM, we initially created a nomogram to estimate the risk of cuproptosis clusters in 533 blood samples from patients with major burns (**Figure 10A**). The predictive efficiency of the nomogram model was assessed using calibration and DCA. The calibration curve showed minimal discrepancy between the actual major burn clustering risk and the predicted risk (**Figure 10C**), and the DCA demonstrated that our nomogram had high accuracy and could provide guidance for clinical decision-making (**Figure 10B**). Subsequently, we validated our 5-gene prediction model using two blood tissue datasets: one from a normal group and the other from patients with major burns. The ROC curve showed that the AUC of the 5-gene prediction model in the GSE19743 dataset was 0.982 (**Figure 10D**), indicating that our diagnostic model was equally effective in distinguishing patients with major burns from normal individuals. Based on the clinical characteristics, the relationship between major burn injuries (**Figures 11A–E**) and the time of injury was predicted using five genes. LUC7L3 (R=-0.24,P=0.01), LRRC47 (R=-0.23, P=0.004), and USPL1 (R=-0.29,P=0.0018) showed a negative correlation with the time of burninjury (**Figures 11A, C, E**).

### 3.9 Differential expression of the signature genes

**Figure 12:**
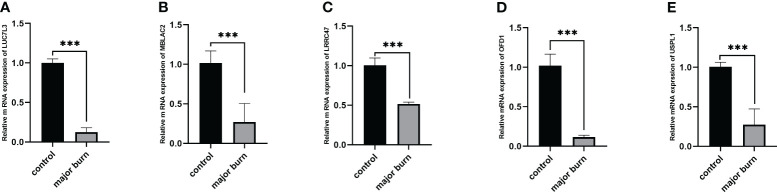
Expression analysis of 5 GENES in major burns and controls. **(A–E)** Differences in mRNA levels of 5 genes between the major burns and control groups. ***p < 0.001.

To further validate this finding, we used RT-qPCR to identify the relative mRNA expression levels of the five target genes in six pairs of samples from normal and major burn conditions. The results showed Significant differences were observed in the expression levels of LUC7L3, MBLAC2, LRRC47, OFD1, and USPL1 between the normal and major burn groups, with decreased expression observed in the major burn groups (**Figure 12**).

## 4 Discussion

Major burns involving large areas can lead to shock, infection, sepsis, and even death (29). Despite improvements in burn wound care and infection control measures over the past few decades, the mortality rate of critically ill patients with burns remains high and challenging to reduce (30). The treatment of major burns mainly focuses on symptomatic management and empirical medication, and it is difficult to assess patient prognosis. Therefore, it is particularly important to understand the specific molecular clusters and personalized treatments related to changes in the body after burns. Cuproptosis is a Cu-dependent form of cell death associated with mitochondrial metabolism. Multiple studies have shown a close association between cardioproptosis and the occurrence and progression of various diseases, such as atherosclerosis, pulmonary hypertension, epilepsy, and tumors (31–33). Additionally, patients with sepsis may have elevated levels of Cu ions in their body.

This study investigated the expression profiles of CRGs in the blood of healthy individuals and patients with major burns for the first time. Compared with normal subjects, 15 CRGs were found to be abnormal in patients with major burns, including ATP7A, GLS, LIAS, DBT, PDHB, FDX1, LIPT1, CDKN2A, PDHA1, DLST, SLC31A1, DLD, NLRP3, ATP7B, and MTF1. This suggests that CRGs play an important role under post-burn conditions. Subsequently, we analyzed the correlation between CRGs to elucidate the relationship between cuproptosis and major burns. Correlation analysis revealed significant synergistic or antagonistic effects among the cuproptosis regulatory factors.

In the analysis of immune cell infiltration among the 22 cell types, the expression levels of B cells, plasma cells, regulatory T cells (Tregs), monocytes, macrophages M0, eosinophils, and neutrophils were elevated in burn patients. The imbalance between hyperimmune response and immunosuppression contributes to increased mortality in critically ill patients (34). Aberrant CD4^+^ and CD8^+^ T-cell responses are major components of acquired immune response dysregulation, and immune dysfunction in Tregs contributes to pathogenesis (35). It has been reported that improving the heterogeneity characteristics of Tregs through intervention strategies can improve the prognosis of sepsis (36).

Additionally, based on the expression profiles of the 15 CRGs, we utilized unsupervised clustering analysis to elucidate distinct copper imbalance regulatory patterns based on the CRGs expression landscape in patients with major burns and identified two distinct copper imbalance-related clusters. In Cluster2, there had elevated immune scores and relatively high levels of immune infiltration. Expression of ATP7B, ATP7A, SLC31A1, FDX1, LIAS, LIPT1, DLD, PDHA1, PDHB, GLS and DBT was upregulated in Cluster2. Moreover, immune cell infiltration analysis revealed a high expression of B cells naïve, T cells CD4 naïve, T cell CD4 memory resting, T cells CD4 memory activated, T cells gamma delta, resting NK cells resting, and Neutrophils in Cluster2. KEGG pathway analysis revealed that Cluster2 was primarily enriched in biological processes related to adherens junctions, long-term potentiation, and insulin signaling pathways, which are associated with post-burn hyperglycemia (37, 38). On the other hand, Cluster1 was characterized by ribosome and fatty acid metabolism, suggesting an impact of major burns on multiple cellular metabolic pathways (34).

In recent years, machine learning models based on demographic and imaging indicators have been increasingly used to predict disease prevalence. Studies have shown that a multifactor analysis, which considers the relationships between variables, has lower error rates and more reliable results than a single-factor analysis (39). In this study, we assessed and compared the predictive performance of four specifically chosen machine learning classifiers (RF, SVM, GLM, and XGB) based on cluster-specific GLM expression profiles and established a GLM-based predictive model that demonstrated the highest predictive capability (AUC = 0.989). This indicates that machine learning based on GLM performs satisfactorily in predicting major burn-induced physiological changes in the body. Subsequently, five important variables (LUC7L3, MBLAC2, LRRC47, OFD1, and USPL1) were selected to construct a GLM model based on these five genes. LUC7L3 is a splicing factor containing arginine- and glutamic acid-rich (RE) and arginine- and serine-rich (RS) domains. Currently, there is limited research on LUC7L3, although it is known that LUC7L3 is upregulated in heart failure (40). Knockdown of LUC7L3 has been identified as a target gene of miR-370-5p. The alternate expression of LUC7L3 reverses the regulatory effect of miR-370-5p on the phenotype of breast cancer cells, making it a potential new target for cancer treatment (41). LUC7L3 also inhibits HBV virus replication (42). However, the role of LUC7L3 in severe burns and the development of drug targets still require further research. MBLAC2 stands for Metallo-β-lactamase domain-containing protein 2, which has potent acyl-CoA thioesterase activity *in vitro* (43). There are limited reports on MBLAC, which may be associated with myoclonic epilepsy (44). It is worth noting that MBLAC2 is a common off-target of hydroxamic acid drugs, indicating that MBLAC2 has potential for drug development (45). LRRC47 contains leucine-rich repeat sequences, which are structural motifs involved in protein-protein interactions. LRRC47 plays a role in various cellular processes, including cell adhesion, signal transduction, and immune response regulation (46). It interacts significantly with PDZ-binding kinase (PBK) and influences the prognosis and immune infiltration of liver cancer (47). It is closely related to the growth of prostate cancer cells and shows good therapeutic potential (48). The OFD1 gene (Oral-Facial-Digital Syndrome 1 gene) is responsible for Oral-Facial-Digital Syndrome type 1. OFD1 is located on the X chromosome in humans and encodes OFD1 plays crucial roles in early embryonic development. Mutations in OFD1 can lead to OFD1 syndrome, which is a rare genetic disorder. In addition to its role in early embryonic development, OFD1 plays an important role in cellular processes such as autophagy (49–51). In particular, OFD1 is the first example of a ciliopathy protein that controls protein expression and autophagy/proteasome degradation, providing directions for the treatment of various diseases (52). USPL1 (Ubiquitin-Specific Peptidase-Like 1) is a gene that encodes a protein. USPL1 belongs to the protease family and its structure is similar to that of specific proteases. It plays a role in cellular deubiquitination by removing ubiquitin tags from proteins. Ubiquitination is an important cellular mechanism that regulates protein stability, function, and subcellular localization (53). USPL1 participates in various biological processes including DNA repair, cell cycle regulation, and signal transduction. Mutations or abnormal expression of this gene are associated with certain diseases, such as tumors, neurodegenerative diseases, and immune system disorders (54–56). USPL1 has potential for research in various diseases. After major burns, the internal environment of the body undergoes significant changes, making it crucial to predict the prognosis and treatment. Therefore, we conducted a correlation analysis of the time of injury in patients with major burns by using five predictive genes. Although our study did not directly demonstrate the relationship between the five core genes and the severity and prognosis of burns, such as infection, respiratory failure, organ dysfunction, and wound healing, we proved that the levels of LUC7L3, LRRC47, and USPL1 were negatively correlated with the course of the disease (p<0.05). However, the course of the disease is often positively correlated with the severity of the disease. The complex relationship between these genes and the disease, as well as their mechanisms, require further analysis and experimental research. In conclusion, the GLM based on these five genes is a satisfactory indicator for assessing the pathological outcomes of patients with major burns. This study contributes to predicting the prognosis and treatment targets for patients with severe burns, laying the foundation for their clinical application, and has research potential.

This study has some limitations. First, this study was based on a comprehensive bioinformatics analysis and preliminary experimental validation. Further clinical or experimental evaluations with larger sample sizes are needed to validate the expression levels of CRGs. Therefore, additional clinical features are required to improve the performance and robustness of the prediction model. Furthermore, improving the accuracy of cuproptosis-related clusters requires additional samples from major burns. Finally, the potential correlation between CRGs and immune cell infiltration is not fully understood and requires further investigation.

## 5 Conclusions

Our study revealed the relevance between CRGs and infiltrating immune cells, highlighted significant immune heterogeneity among major burn patients in different cuproptosis-related clusters. The GLM model based on five genes emerged as the best machine learning model for evaluating major burn patients. We provided previously unrecorded evidence and conducted experimental validation to demonstrate the role of cuproptosis in major burns.

## Data availability statement

The datasets presented in this study can be found in online repositories. The names of the repository/repositories and accession number(s) can be found in the article/supplementary material.

## Ethics statement

The studies involving humans were approved by Committees of The First Affiliated Hospital of Nanchang University. The studies were conducted in accordance with the local legislation and institutional requirements. The participants provided their written informed consent to participate in this study.

## Author contributions

XW: Conceptualization, Data curation, Formal analysis, Investigation, Methodology, Software, Validation, Visualization, Writing – original draft, Writing – review & editing. ZX: Validation, Writing – review & editing. WH: Validation, Writing – review & editing. XL: Data curation, Writing – review & editing. GY: Validation, Writing – review & editing. ZJ: Validation, Writing – review & editing. LJ: Validation, Writing – review & editing. SH: Validation, Writing – review & editing. ZF: Supervision, Writing – review & editing, Funding acquisition, Methodology. FZ: Supervision, Writing – review & editing, Formal analysis.
